# Oral glucose solution for the treatment of persistent asymptomatic neonatal hypoglycemia: a randomized, double-blind, placebo-controlled trial

**DOI:** 10.1007/s00431-026-07427-y

**Published:** 2026-09-29

**Authors:** Ratchada Kitsommart, Thananjit Petchkum, Buranee Yangthara, Pitiporn Siripattanapipong, Bosco Paes

**Affiliations:** 1 Division of Neonatology, Department of Pediatrics, Faculty of Medicine Siriraj Hospital, Mahidol University, Bangkok, Thailand; 2 Department of Pediatrics, Bhumibol Adulyadej Hospital, Royal Thai Air Force, Bangkok, Thailand; 3 Division of Neonatology, Department of Pediatrics, McMaster University, Hamilton Health Sciences, Hamilton, ON Canada

**Keywords:** Asymptomatic, Intravenous line, Hypoglycemia, Oral dextrose, Postnatal, Neonate

## Abstract

To evaluate the efficacy of 50% dextrose solution (D50W) for treating persistent asymptomatic postnatal hypoglycemia in at-risk late preterm and term neonates and the effect on intravenous (IV) dextrose therapy. A multicenter, randomized, double-blind, placebo-controlled trial conducted at two high-risk nurseries in Bangkok, Thailand. At-risk neonates ≥ 34 weeks’ gestation with persistent asymptomatic hypoglycemia, defined as a blood glucose level that remained below the postnatal, age-dependent protocol threshold 30 min after the first feeding, were randomized to receive either buccal D50W (200 mg/kg, 0.4 mL/kg; OG group) or placebo, followed by enteral feeding. The primary outcome was the proportion of infants requiring IV dextrose. Secondary outcomes included changes in blood glucose (BG) levels and hyperglycemia. Four-hundred infants were enrolled (OG: *n* = 205; placebo: *n* = 195). Median birthweight was comparable between groups. Pre-treatment BG levels were similar (OG 37.0 [33.0, 39.0] mg/dL vs. placebo 37.0 [33.0, 39.0] mg/dL; *p* = 0.47). The proportion requiring IV dextrose was lower in the OG group but did not differ significantly (4.9% vs. 8.7%; *p* = 0.18). Median BG increment post-treatment was significantly greater in the OG group than in the placebo group (28.0 [18.0,40.0] vs. 22.5 [14.0, 35.0] mg/dL; *p* < 0.01). Hyperglycemia did not occur in either group. Linear mixed-effects analysis demonstrated that D50W significantly increased BG concentrations, whereas increasing postnatal age and small- or large-for-gestational-age status were associated with smaller BG responses. *Conclusions*: Buccal D50W administration did not significantly reduce the need for IV dextrose therapy in asymptomatic neonates with hypoglycemia, although it resulted in a greater increase in BG levels than placebo. Further studies are needed to determine its clinical role in the management of neonatal hypoglycemia.

**Table:** 

What is Known: • Oral 40% dextrose gel is recommended for asymptomatic neonatal hypoglycemia but is unavailable in many under-resourced healthcare settings. • Intravenous dextrose is effective but may disrupt mother-infant contact and breastfeeding.	
What is New: • Buccal 50% dextrose increased blood glucose more than placebo but did not reduce IV dextrose use. • No episodes of hyperglycemia, choking, vomiting, or mucosal irritation were observed in either group during routine clinical monitoring.

*Trial registration*: Thai Clinical Trials Registry (TCTR), TCTR20181204005, registered on December 4, 2018. https://www.thaiclinicaltrials.org/show/TCTR20181204005

**Supplementary information:**

The online version contains supplementary material available at https://doi.org/10.1007/s00431-026-07427-y.

## Introduction

Neonatal hypoglycemia is a common complication during the postnatal period, affecting up to 51% of at-risk neonates [1, 2]. Severe or recurrent hypoglycemia can disrupt physiological functions, particularly in the nervous system, leading to abnormal clinical manifestations and increased risk of future developmental delay [3, 4]. Therefore, neonates at risk of hypoglycemia or those exhibiting abnormal symptoms require close monitoring of blood glucose (BG) levels and appropriate management [5].

Treatment of hypoglycemia depends on clinical presentation and BG levels. Intravenous (IV) glucose is indicated for symptomatic or severe hypoglycemia but requires vascular access, which may cause pain, potentially increases risk of infection, disrupts breastfeeding and maternal-infant bonding, and contributes to BG fluctuations or hyperglycemia [6–9].

Currently, oral 40% dextrose gel is widely utilized for the prevention or treatment of postnatal hypoglycemia in at-risk neonates [10–13]. Oral dextrose gel reduces both the need for further hypoglycemia correction and IV dextrose administration [12, 14], and is recommended in conjunction with breastfeeding for neonates with low or borderline BG levels [10, 15]. Despite its benefits, commercially prepared oral dextrose gel is not universally available in many resource limited settings [16]. Given that neonates require small doses of oral glucose gel, its use raises concerns about the uniformity of glucose distribution within the gel, potentially leading to inconsistent efficacy [17, 18]. Additionally, gel administration poses challenges, such as difficulty in controlling precise dosage during buccal application, maximum recommended dosage in 48 h, potential oral leakage, and undue messiness during treatment.

Evidence gleaned from adults, indicates that highly concentrated oral glucose solutions are effective for the treatment of mild hypoglycemia [19, 20]. We adopted this approach in neonates to evaluate whether buccal administration of D50W, in addition to a standardized feeding protocol, could reduce the need for IV dextrose therapy in neonates with persistent asymptomatic hypoglycemia. We hypothesized that D50W plus feeding would reduce the proportion of infants subsequently requiring IV dextrose therapy compared with placebo plus the standardized feeding protocol. The primary objective was to compare the proportion of infants subsequently requiring IV dextrose therapy between those receiving D50W plus feeding and those receiving placebo plus the same feeding protocol. Secondary objectives were to compare the changes in BG levels and the incidence of hyperglycemia between the two groups.

## Material and methods

### Trial design and setting

A multicenter, prospective, randomized, double-blind, placebo-controlled trial was conducted at the two high-risk nurseries, Siriraj and Bhumibol Adulyadej Hospitals in Bangkok, Thailand. Recruitment at Siriraj Hospital began on December 5, 2018, and continued until January 6, 2026, while recruitment at Bhumibol Adulyadej Hospital commenced on June 17, 2020, and continued until June 6, 2025.

Both nurseries admit neonates who have mild respiratory and hemodynamic instability, or are at risk for or diagnosed with hypoglycemia based on a standardized postnatal hypoglycemia care protocol, consistent with the 2011 American Academy of Pediatrics (AAP) guidelines [21].

BG monitoring is routinely performed on neonates born at ≥ 34 weeks of gestation who are identified as at-risk or exhibit symptoms aligned with hypoglycemia. Neonates presenting with postnatal symptoms and a BG level below 40 mg/dL receive IV dextrose therapy, while asymptomatic neonates are managed with early feeding of infant formula (5 mL/kg), followed by reassessment of BG levels. Acceptable glucose thresholds were defined as BG ≥ 40 mg/dL in the first four hours of life and BG ≥ 45 mg/dL from 4–24 h of age, with IV dextrose administered if BG levels fell below 25 mg/dL or 35 mg/dL, respectively.

### Participants

Infants with asymptomatic hypoglycemia within the first 48 h of life were eligible for the study. Inclusion criteria encompassed at-risk neonates, including preterm infants (gestational age [GA], 34–37 weeks), infants of diabetic mothers, small-for-gestational-age neonates (SGA; birth weight [BW] < 10th percentile), large-for-gestational-age neonates (LGA; BW > 90th percentile), and neonates with BW < 2,500 g or > 4,000 g who demonstrated asymptomatic hypoglycemia despite early feeding, with BG levels measured 30 min after feeding. Hypoglycemia was defined as a BG level < 40 mg/dL within the first 4 h of life or < 45 mg/dL between 4 to 48 h of life. Neonates with a BG level < 25 mg/dL in the first 4 h or < 35 mg/dL during the 4- to 48-h period, were treated with IV dextrose and were excluded. Potential treatment-related adverse events, including choking, vomiting, and clinically apparent oral or mucosal irritation, were monitored by nursing staff during routine nursery care after administration of the allocated solution. Adverse events were recorded when clinically observed. Hyperglycemia was defined as a BG level > 180 mg/dL. Additionally, neonates requiring IV fluids for reasons other than hypoglycemia, those kept “*nil per os”* for procedures, and those with neurological impairment were excluded.

### Interventions

The oral glucose group (OG group) received a 50% dextrose solution (D50W), while the placebo group received sterile water. Each solution was administered by slow application to the buccal mucosa over 1–2 min, at a dose of 0.4 mL/kg (200 mg glucose/kg) followed by cup feeding. Nursing staff were trained to clean the buccal mucosa prior to application and to administer the solution gently. BG levels were re-evaluated 30 min after administration, and the assigned solution could be re-administered if hypoglycemia persisted. A maximum of six doses was allowed within the first 48 h of life. If BG levels returned to the acceptable range, subjects were monitored with pre-feeding BG checks every 2–4 h until stability was maintained for at least 24 h. The feeding protocol consisted of feeds every 3 h of 5 mL/kg during the first day of life, with daily increases of 30 mL/kg/day thereafter. Breast milk was provided when available, and formula was used when breast milk was unavailable. Direct breastfeeding was provided in accordance with the unit’s breastfeeding policy.

### Randomization and allocation

Block randomization was performed with block sizes of 2–4, assigning neonates in a 1:1 ratio to either the treatment or placebo group within each center. For multiple gestation pregnancies in which more than one infant met eligibility criteria, each eligible infant was randomized independently. This approach was chosen because, despite sharing maternal and gestational characteristics, co-twins can differ in factors that may influence the physiological response to hypoglycemia and its treatment, including intrauterine growth status and birth weight.

Allocation concealment was ensured through sequentially numbered opaque envelopes prepared according to a pre-generated randomization list.

Both solutions were prepared by the General Pharmaceutical Production Division of the Pharmacy Department at Siriraj Hospital, packaged identically, and were colorless and indistinguishable. The pharmacist responsible for preparing the solutions was not involved in participant care or any other aspects of the study.

### Outcomes

The primary outcome was the proportion of infants requiring IV dextrose therapy during the study period. Secondary outcomes included the change in BG level following administration of the allocated solution and adverse events including hyperglycemia, choking, vomiting, and clinically apparent oral or mucosal irritation.

### Glucose monitoring and measurement

Glucose monitoring was performed according to the neonatal standard care protocols. BG levels were measured using heel stick or venous blood samples applied to StatStrip™ glucose meters, which utilize the modified glucose oxidase method, or Accu-Chek™ Inform II devices (Roche, Mannheim, Germany), which use the glucose dehydrogenase method. If the point-of-care (POC) glucose measurement was < 50 mg/dL, a confirmatory venous blood sample was drawn and analyzed in the central laboratory using the hexokinase method. Confirmatory testing was conducted with a Roche Modular P 800 analyzer (Roche Diagnostics, Thailand, Co., Ltd).

### Sample size

The sample size was estimated based on previous observations from the Siriraj high-risk nursery that 26% of at-risk neonates with asymptomatic hypoglycemia required IV dextrose. According to Harris et al., [22] the proportion of neonates receiving IV dextrose was 7% in the glucose gel group compared to 14% in the placebo group, yielding a risk ratio of 0.5. These proportions were used to estimate the expected treatment effect and calculate the sample size using the two-independent-proportion method, with a type I error of 0.05, a type II error of 0.2, which yielded 160 neonates per group. The target sample size was increased to 384 participants to account for a 20% potential loss of evaluable participants and was ultimately set at 400 to ensure adequate enrollment.

### Statistical analysis

All statistical analyses and graphical visualizations were performed using R software (version 4.4.3). Demographic data were analyzed according to the type and distribution of variables. Continuous variables are presented as median [25th, 75th percentile; P25, P75], and categorical variables as number (percentage). Analyses were conducted on an intention-to-treat basis. Comparisons of categorical variables between groups were performed using the chi-square or Fisher’s exact test, as appropriate, and continuous variables were compared using the Mann–Whitney U test. Relative risks with 95% confidence intervals for IV dextrose requirement were calculated using the Wald method via the *epitools* package.

For the primary outcome of IV dextrose requirement, logistic regression adjusted for recruitment site was used, with results presented as an adjusted odds ratio (OR) with 95% confidence interval (95%CI). For BG increment following administration of the allocated solution during hypoglycemic episodes, a linear mixed-effects model with a random intercept for each infant was used, fitted using the *lmerTest* package in R, to account for infants who contributed more than one hypoglycemic episode. Covariates in this model included solution type, postnatal age, recruitment site, gestational age (mean-centered), birthweight (mean-centered, per 100-g increment), small-for-gestational age (SGA), large-for-gestational age (LGA), infant of a diabetic mother, and the interaction between solution type and postnatal age. Results are reported as coefficients with 95% confidence intervals. A *p*-value less than 0.05 was considered statistically significant. A figure demonstrating the trend of BG over time was generated using the *ggplot2* package.

### Ethics and trial registration

The study protocol received approval from the institutional review boards of both participating centers (Siriraj COA no. Si 523/2018) and was registered with the Thai Clinical Trials Registry (TCTR20181204005: https://www.thaiclinicaltrials.org/show/TCTR20181204005). Written parental consent was obtained from all participants prenatally or during the early postnatal period, prior to infant recruitment.

## Results

### Participant flow and baseline characteristics

**Fig. 1:**
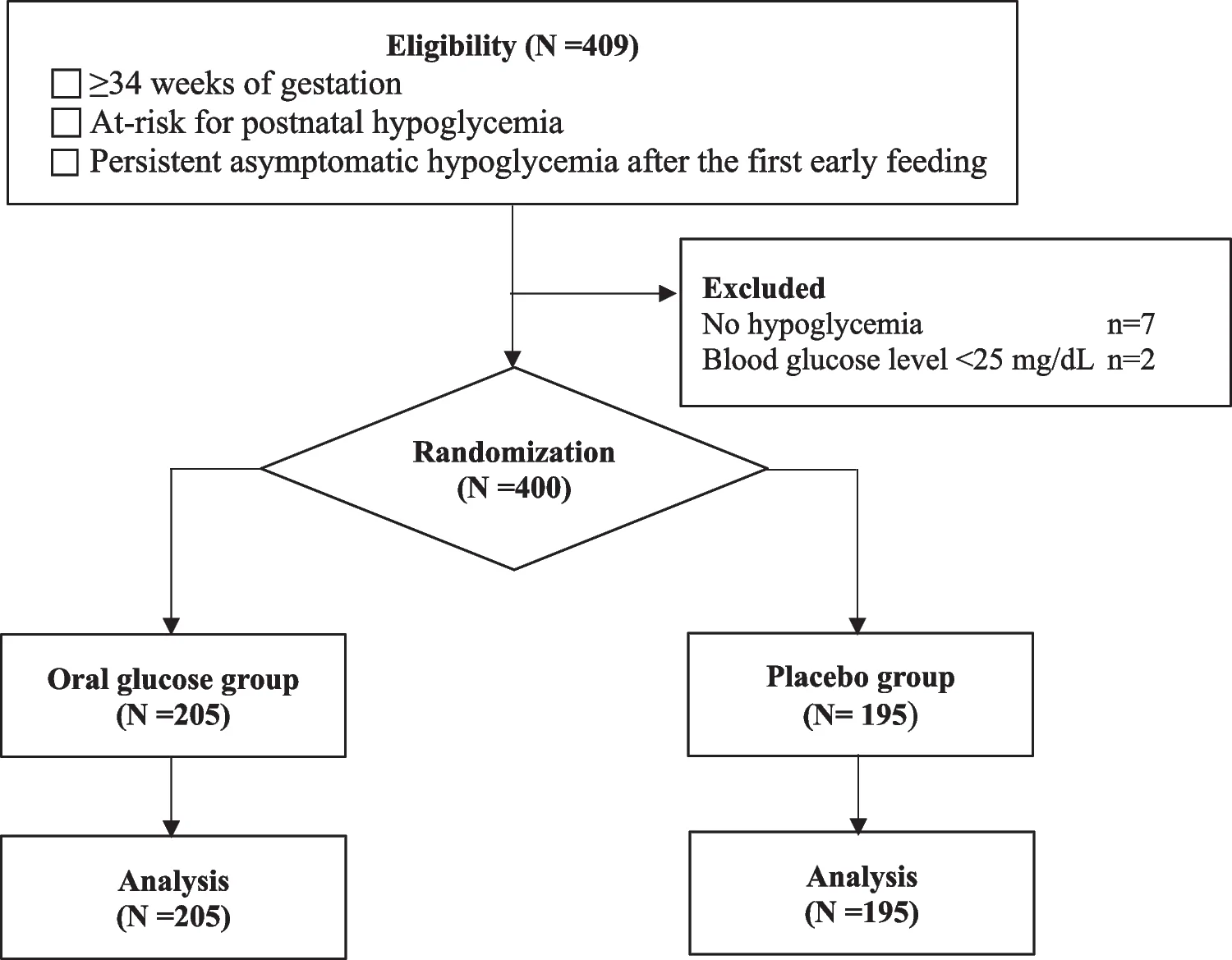
Study flow diagram

A total of 400 infants with persistent, asymptomatic postnatal hypoglycemia after early feeding were enrolled, with 205 infants allocated to the OG group and 195 infants to the placebo group. Figure 1 outlines the study flow. Twelve infants (3.0%) were born from six twin pregnancies in which both siblings met eligibility criteria and were independently randomized. Treatment allocation differed between co-twins in four of the six pairs (66.7%). The median postnatal age at enrollment was 2.0 [1.3, 5.0] hours. Baseline maternal and neonatal characteristics were generally comparable between groups, except that the rate of caesarean delivery was significantly higher in the OG group compared with the placebo group (69.2% vs. 58.0%, *p* = 0.03) (Table 1 and Supplementary Table S1). The two centers were similar with respect to GA, birth weight, sex, SGA, and LGA status, although maternal age and postnatal age at diagnosis of hypoglycemia differed between centers (Supplementary Table S2).

**Table 1:** Infants’ baseline demographic characteristics (*N* = 400)

	50% Dextrose solution (*n* = 205)	Placebo solution (*n* = 195)	p*
Male sex	93 (45.4)	106 (54.4)	0.09
Gestational age (weeks)	37 [35, 38]	37 [36, 38]	0.26
34–37	87 (42.4)	75 (38.5)	0.48
Maternal diabetes	81/205 (40.3)	92/195 (47.7)	0.17
Birth weight (g)	2520 [2260, 3080]	2690 [2335,3205]	0.09
Small-for-gestational age	29 (14.1)	23 (11.8)	0.58
Large-for-gestational age	19 (9.3)	18 (9.2)	1.00
5-min Apgar score	10 [9, 10]	10 [9, 10]	0.84
Postnatal age at diagnosis of hypoglycemia (hour)	2.0 [1.3, 5.0]	2.0 [1.3, 5.0]	0.84

Data are presented in number (percentage) or median [25th,75th percentile]

*p-value less than 0.05 is considered statistically significant

### Primary and secondary outcomes

Infants’ BG levels and hospital outcomes are presented in Table 2. At enrollment, median first postnatal BG levels were similar between the groups (37.0 [33.0, 39.0] vs. 37.0 [33.0, 39.0] mg/dL in the OG group and the placebo group, respectively; *p* = 0.47). The proportion of infants requiring IV dextrose therapy was lower in the OG group (4.9%) than in the placebo group (8.7%), though the difference was not statistically significant (*p* = 0.18; RR, 0.62; 95% CI, 0.30–1.28). The rate of IV dextrose therapy did not differ significantly between centers (5.9% vs. 12%, *p* = 0.10; Supplementary Table S2) and after adjustment, recruitment center was not significantly associated with IV dextrose requirement (adjusted OR, 2.20; 95% CI, 0.80–5.59; *p* = 0.11). For the secondary outcomes, median incremental increase in BG levels was greater in the OG group than in the placebo group (28.0 [18.0, 40.0] vs. 22.5 [14.0, 35.0] mg/dL; *p* < 0.01). The rate of infants experiencing more than one hypoglycemic episode during the study period was similar between groups (13.7% vs. 13.8%; *p* = 1.00). No cases of hyperglycemia, choking, or vomiting were observed in either group. The maximum number of allocated solution doses administered were 3 and 5 in the OG and placebo groups, respectively (median doses were 1 [1, 1] in both groups, *p* = 0.39). Length of hospital stay did not differ significantly between groups (median 5 [4, 7] days in both groups, *p* = 0.06); Table 2).

**Table 2:** Comparison of clinical outcomes between the oral glucose and placebo groups (*N* = 400)

	50% Dextrose solution (*n* = 205)	Placebo solution (*n* = 195)	p*
First BG level (mg/dL)	37.0 [33.0, 39.0]	37.0 [33.0, 39.0]	0.47
BG levels after the first treatment (mg/dL)	68.0 [56.8, 77.3]	60.0 [51.0, 74.0]	< 0.01*
< 40	7/205 (3.4)	9/195 (4.6)	0.73
< 25	1/205 (0.5)	1/195 (0.5)	1.00
Incremental BG level after the first treatment (mg/dL)	30.0 [20.0, 42.0]	23.0 [15.0, 36.0]	< 0.01*
Incremental BG level after any treatment^†^ (mg/dL)	28.0 [18.0,40.0]	22.5 [14.0, 35.0]	< 0.01*
Number of solution doses needed	1 [1, 1]	1 [1, 1]	0.39
≥ 2	23 (11.2)	17 (8.9)	0.48
Intravenous dextrose therapy	10 (4.9)	17(8.7)	0.18
Hyperglycemia (> 180 mg/dL)	0 [0, 0]	0 [0, 0]	1.00
Duration of birth hospitalization (days)	5 [4, 7]	5 [4, 7]	0.06

Data are presented in number (percentage) or median [25th,75th percentile]

†Two post-treatment BG measurements were not performed in the placebo group after the 2nd and 3rd doses

^*^p-value less than 0.05 is considered statistically significant

*BG* blood glucose

### BG response according to postnatal age

**Fig. 2:**
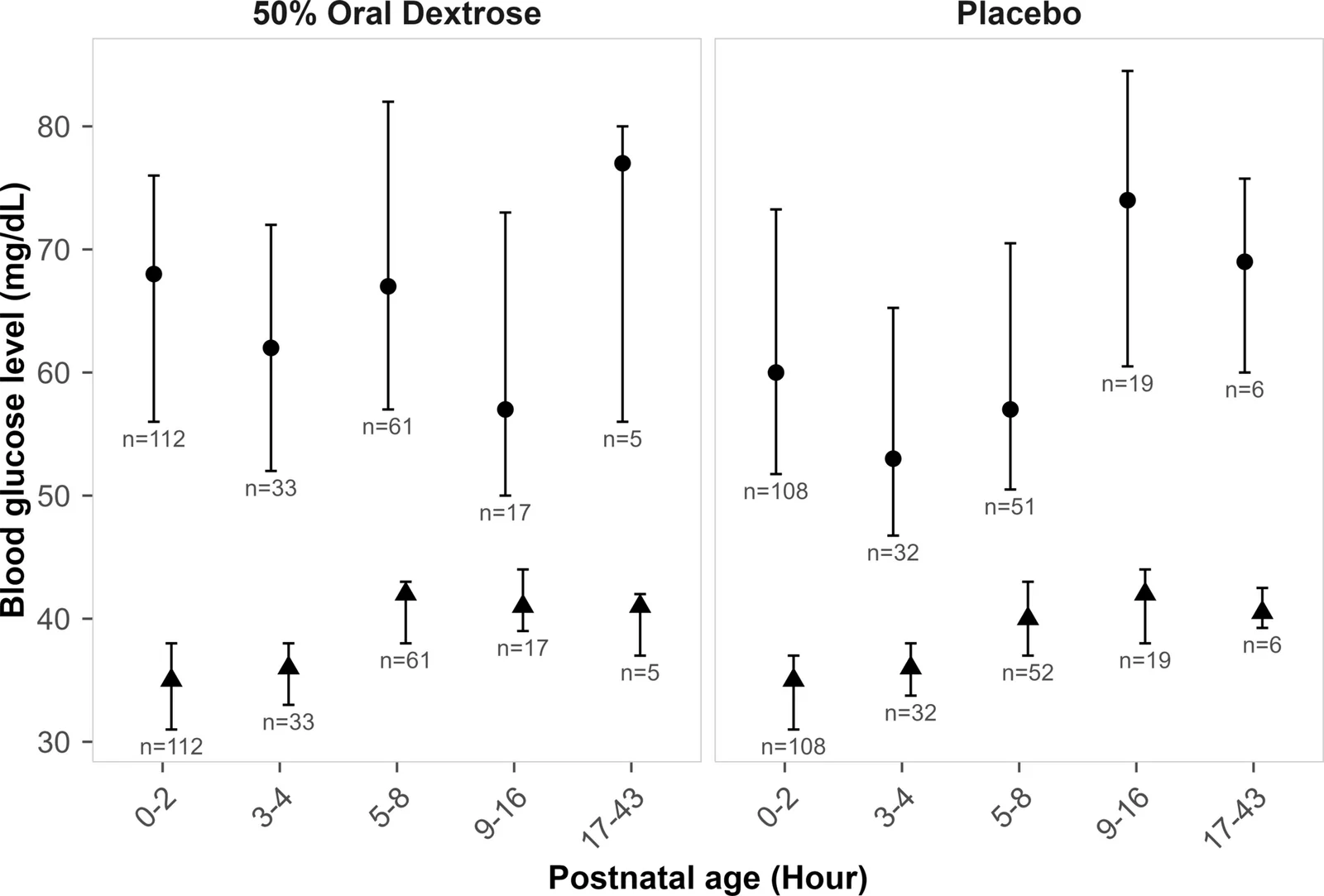
Blood glucose levels before and after enteral feedings with the oral solutions Footnote: Data are presented as median [25th, 75th percentiles]; error bars represent the interquartile range. Triangles indicate blood glucose levels before, and circles after, enteral feeding with the allocated solution

Figure 2 presents BG levels before and after enteral feeding with the allocated solution across postnatal age. Post-treatment BG levels were consistently higher than pre-treatment levels across all postnatal age intervals studied. The magnitude of BG increase appeared greater in the OG group than in the placebo group during the earlier intervals, though the increase was numerically greater in the placebo group at 9 to 16 h. Data in the widest and latest interval (17 to 43 h) were relatively limited (*n* = 5 in the OG group, *n* = 6 in the placebo group).

### Factors associated with BG increment

A total of 444 hypoglycemic episodes across 398 infants were analyzed using a linear mixed-effects model to identify factors associated with BG increments following administration of the allocated solution (Table 3). Two post-treatment BG measurements were not performed in the placebo group after the 2nd and 3rd doses; therefore, these infants were excluded from the analysis.). After adjustment, recruitment center was not significantly associated with BG increment (adjusted difference, −1.48 mg/dL; 95%CI, −5.6, 2.6; *p* = 0.477). Administration of oral D50W was associated with a significantly greater BG increase (adjusted difference, 8.38 mg/dL; 95% CI, 4.5, 12.0; *p* < 0.001). Each 1-h increase in postnatal age was not significantly associated with BG increment (adjusted difference, 0.19 mg/dL; 95% CI, −0.27, 0.60; *p* = 0.455). SGA and LGA infants demonstrated significantly lower BG increments than non-SGA and non-LGA infants, respectively (adjusted difference, −5.77; 95%CI, −11.00, −0.59; *p* = 0.029 and −7.06; 95%CI, −14.00, −0.31, *p* = 0.040, respectively). Neither birthweight nor GA alone showed a significant association with BG increment. However, a significant interaction was observed between treatment group and postnatal age (interaction coefficient −0.88; 95% CI, −1.5, −0.24; *p* = 0.007), indicating that the adjusted difference in BG increment between treatment groups narrowed with increasing postnatal age. Model-predicted BG increments and adjusted treatment differences at representative postnatal ages are presented in Supplementary Table S3.

**Table 3:** Factors associated with incremental blood glucose levels using linear mixed effect model (*N* = 444 hypoglycemic episodes across 398 infants)

Factors	Blood glucose increment (mg/dL)	
	Coefficient (95% confidence interval)	p*
Intercept	27 (23, 30)	< 0.001*
50% oral dextrose solution	8.38 (4.5, 12.0)	< 0.001*
Postnatal age in the placebo group (hour)	0.17 (−0.27. 0.60)	0.455
Recruitment site (Bhumibol Adulyadej vs. Siriraj)	−1.48 (−5.6, 2.6)	0.477
Gestational age, centered (weeks)	1.04 (−0.11, 2.20)	0.075
Birthweight, centered (every 100-g increment)	−0.00 (−0.4, 0.4)	0.998
Small-for-gestational age	−5.77 (−11.00, −0.59)	0.029*
Large-for-gestational age	−7.06 (−14.00, −0.31)	0.040*
Infant of diabetic mother	−0.65 (−4.1, 2.8)	0.710
Postnatal age-solution interaction	−0.88 (−1.5, −0.24)	0.007*

Random effects: Residual variance (σ^2^) = 190.80; random intercept variance (τ00) = 33.89; ICC = 0.15; marginal R^2^ = 0.068; conditional R^2^ = 0.208; RMSE = 12.6 mg/dL

Linear mixed-effects model (dependent variable: blood glucose increments following administration of allocated solution), with a random intercept per infant. Small-for-gestational age (SGA) was compared against non-SGA infants; large-for-gestational age (LGA) was compared against non-LGA infants; infant of diabetic mother was compared against infants of non-diabetic mothers. Two post-treatment BG measurements were not performed in the placebo group after the 2nd and 3rd doses. *p-value less than 0.05 is considered statistically significant

## Discussion

For asymptomatic neonatal hypoglycemia, oral D50W (200 mg/kg), consistent with established dosing, was administered via the buccal mucosa to maximize absorption [22]. We chose to administer the intervention only after the first early feeding when BG levels remained below the acceptable threshold. Of note, feeding alone can often raise BG levels [23], and early feeding avoided unnecessary exposure to dextrose in otherwise asymptomatic infants which may reduce the likelihood of maternal-infant separation during the management of hypoglycemia as opposed to symptomatic infants who unquestionably require IV therapy.

Although the point estimate favored the OG group, with a relative risk of 0.62, the 95% confidence interval was wide (0.30–1.28), indicating limited precision in the estimated treatment effect. The number of infants requiring IV dextrose was lower than anticipated in the sample size assumptions, resulting in a relatively small number of outcome events and consequently limiting the precision of the estimated treatment effect. Nevertheless, the OG group achieved a significantly greater increase in BG levels, indicating a greater acute physiological response to oral D50W administration. The magnitude of the BG response can be contextualized with findings from previous studies of oral glucose gel, although differences in study populations, feeding practices, glucose thresholds, timing of BG measurements, and outcome definitions limit direct comparisons. Our findings are comparable with those of Harris et al. [23] in which infants received similar feeding volumes (5 vs. 4.5 mL/kg) and had comparable pre-treatment BG levels (37.0 vs. 41.4 mg/dL). However, in the present study we observed a median BG increment of 28.0 mg/dL after oral dextrose solution administration followed by formula feeding, which was even more pronounced after the first treatment (30.0 mg/dL). This compares with a BG increment of 15.5 mg/dL following dextrose gel and formula feeding in the Harris study [23] and 24.0 mg/dL in the study by Gupta et al. (2022) [24]. Although formula feeding alone also increases BG levels, the placebo group demonstrated a median BG increase of 22.5 [14.0, 35.0] mg/dL, highlighting the important contribution of enteral feeding and transitional physiology to the observed rise in BG. The corresponding increase in the OG group was 28.0 [18.0, 40.0] mg/dL, with a significantly greater increase compared with placebo and the linear mixed-effects model showed that administration of oral D50W was associated with an average 8.38 mg/dL greater increase in BG per hypoglycemic episode compared with placebo. These findings suggest that oral dextrose solution contributed an additional increase in BG beyond that achieved with formula feeding alone. A significant interaction between postnatal age and treatment group indicates that the treatment effect of oral D50W diminishes with increasing postnatal age. To further characterize this interaction, we estimated the model-predicted BG increment and treatment difference at representative postnatal ages (Supplementary Table S3). The predicted treatment difference was largest in infants diagnosed shortly after birth and progressively narrowed with increasing postnatal age. This pattern indicates that the biochemical benefit of oral D50W over placebo is most pronounced when hypoglycemia is diagnosed soon after birth, and diminishes in infants diagnosed later in the postnatal period, consistent with Fig. 2.

Feeding alone contributed substantially to the improvement in BG levels in some asymptomatic neonates, and oral D50W provided an additional glycemic effect. However, given the low number of infants requiring IV dextrose, whether this additional increase in BG translates into a clinically meaningful reduction in treatment failure or other clinical outcomes could not be determined. Moreover, despite the greater acute glycemic response in the OG group, we did not observe a higher proportion of infants experiencing more than one hypoglycemic episode during the study period.

The buccal mucosa, with its rich vascular network, facilitates rapid glucose absorption directly into the bloodstream. Although some glucose may be swallowed and absorbed via the gastrointestinal tract, studies in adults have demonstrated that oral glucose solutions can increase BG levels more effectively than glucose gels [19]. Interestingly, the placebo group in our study demonstrated a BG increment of 22.5 mg/dL, higher than the increment reported by Harris et al. [23] This difference may be explained by the fact that all infants in our study received formula feeding, and the feeding volume was slightly greater than the latter study [25]. The observed rise in BG levels following enteral feeding in both groups supports the role of early feeding as an effective initial intervention for asymptomatic neonatal hypoglycemia [6]. The greater BG increment seen in the OG group is consistent with the additional effect of buccal dextrose absorption. Beyond 24 h of life, BG data were available from only two infants (one in each group), therefore, no meaningful comparisons or conclusions can be made.

The high concentration of D50W facilitates administration in small volumes without displacing milk feeds. Its wide availability and simple administration make it a practical alternative to oral dextrose gel, particularly where commercial preparations are unavailable. In this study, commercially available sterile D50W was divided into small single-use portions by the hospital pharmacy to facilitate administration and ensure identical packaging with placebo for blinding. No additional formulation or compounding of D50W was required. D50W is a standard glucose-containing solution routinely available in hospital settings. However, the feasibility, cost, storage requirements, and regulatory considerations of this approach in resource-limited settings remain to be determined and warrant further implementation research. SGA and LGA infants had smaller BG increments after treatment, possibly reflecting differences in glucose metabolism or relative hyperinsulinism, which may have implications for glucose management in these groups.

While using D50W for buccal administration shows promise, potential concerns remain regarding mucosal irritation, glucose stability, and the risk of hyperglycemia. In this study, no clinically apparent mucosal irritation and no case of hyperglycemia (BG > 180 mg/dL) were observed among infants in the OG group, the majority of whom (88.8%) received only a single dose. However, given the relatively small sample size, predominantly single-dose exposure, and a hyperglycemia threshold that captures only marked elevations in BG, this study cannot exclude uncommon adverse events such as mucosal injury with repeated dosing, dosing errors, aspiration, contamination, or clinically relevant increases in BG that remain below the 180 mg/dL threshold. These findings should therefore not be interpreted as evidence of minimal risk. Larger, systematic safety studies are warranted to rigorously evaluate these specific outcomes, particularly with repeated dosing and in neonates with symptomatic hypoglycemia or those born very preterm.

To our knowledge, this is the first robust clinical trial with a prespecified sample size that explored the use of D50W for the treatment of postnatal asymptomatic hypoglycemia at the same dose commonly used for IV therapy. Although definitions of neonatal hypoglycemia vary slightly across universal protocols, we applied the widely generalizable AAP guideline [21], and the differences between recommendations are minimal and unlikely to affect overall clinical applicability [26].

This study has certain limitations. We had initially planned to assess the postnatal age at which neonates established exclusive breastfeeding as a short-term outcome to determine whether D50W administration might interfere with breastfeeding. However, during the recruitment period, the COVID-19 pandemic led to the implementation of stricter breastfeeding policies, which made breastfeeding initiation more challenging. Consequently, this outcome could not be adequately evaluated. The median number of OG doses administered was one. Nevertheless, even limited exposure to buccal D50W could plausibly affect breastfeeding initiation and maternal-infant contact, and this effect remains unknown and warrants further investigation. In addition, no cases of hyperglycemia were observed, and no infant who achieved an acceptable BG level subsequently fell below the age-specific threshold on scheduled monitoring, supporting the safety of this approach with respect to glycemic control.

The COVID-19 pandemic also transiently impacted admission criteria to the high-risk nursery, which may have affected both eligibility and potential recruitment as reflected by the duration of the study. Furthermore, as noted earlier, the rate of IV dextrose infusion in this study was lower than anticipated based on prior observations used for the sample size calculation, Consequently, fewer outcome events were observed than expected, resulting in limited precision of the estimated treatment effect. In addition, all infants in this study received formula supplementation, which likely contributed to the BG increment observed in both groups. Our findings are therefore most applicable to centers with similar feeding protocols, and whether they extend to exclusively breastfed infants, or to centers prioritizing colostrum, donor milk, rooming-in, or dextrose gel before formula, remains uncertain.

In conclusion, buccal administration of D50W significantly increased BG levels in neonates with persistent asymptomatic hypoglycemia after early feeding. D50W offers a practical and readily available alternative to oral dextrose gel, particularly in low-resource settings where commercial preparations are unavailable. Larger studies are warranted to confirm its effectiveness in reducing IV dextrose use and to evaluate its long-term safety.

## Supplementary information

Below is the link to the electronic supplementary material.

ESM 1 (DOCX 22.8 KB)

## Data Availability

The datasets generated and/or analysed during the current study are available from the corresponding author on reasonable request.
