# Supplementary material for: Oral glucose solution for the treatment of persistent asymptomatic neonatal hypoglycemia: a randomized, double-blind, placebo-controlled trial
Source: Eur J Pediatr. 2026 Sep 29;185(10):783. doi: 10.1007/s00431-026-07427-y (PMC13624021; doi:10.1007/s00431-026-07427-y)
Supplement: Supplementary file 1 [file 431_2026_7427_MOESM1_ESM.docx]

| **Supplementary Table 1 Baseline demographic characteristics of maternal/infant study participants (N=400)** | | | | | |  |
| --- | --- | --- | --- | --- | --- | --- |
|  | | | **50% Dextrose Solution** | **Placebo Solution** | ***p**** | |
| Mothers (N=394) | | | (n = 201) | (n = 193) |  | |
|  | Maternal age (year) | | 32 [27, 36] | 32 [27, 36] | 0.52 | |
|  | Gestational age (weeks) | | 37 [36, 38] | 37 [36, 38] | 0.26 | |
|  |  | 34-37 | 84 (41.8) | 73 (37.8) | 0.48 | |
|  | Diabetes | | 81 (40.3) | 92 (47.7) | 0.17 | |
|  | Hypertension | | 28 (13.9) | 26 (13.5) | 1.00 | |
|  | Antepartum hemorrhage | | 1 (0.5) | 2 (1.0) | 0.62 | |
|  | Abnormal presentation | | 3 (1.5) | 3 (1.6) | 1.00 | |
|  | Multiple gestation | | 2 (0.9) | 4 (2.1) | 0.44 | |
|  | Caesarean section | | 139 (69.2) | 112 (58.0) | 0.03* | |
| Infants (N=400) | | | (n = 205) | (n = 195) |  | |
|  | Male sex | | 93 (45.4) | 106 (54.4) | 0.09 | |
|  | Gestational age (weeks) | | 37 [35, 38] | 37 [36, 38] | 0.26 | |
|  |  | 34-37 | 87 (42.4) | 75 (38.5) | 0.48 | |
|  | Birth weight (g) | | 2520 [2260, 3080] | 2690 [2335,3205] | 0.09 | |
|  |  | Less than 2,500 g | 101 (49.3) | 76 (38.9) | 0.05 | |
|  | Small-for-gestational age | | 29 (14.1) | 23 (11.8) | 0.58 | |
|  | Large-for-gestational age | | 19 (9.3) | 18 (9.2) | 1.00 | |
|  | 1-minute Apgar score | | 8 [8,9] | 9 [8,9] | 0.11 | |
|  | 5-minute Apgar score | | 10 [9, 10] | 10 [9, 10] | 0.84 | |
|  | Postnatal age at diagnosis of hypoglycemia (hour) | | 2.0 [1.3, 5.0] | 2.0 [1.3, 5.0] | 0.84 | |
| Data are presented in number (percentage) or median [25^th^,75^th^ percentile].  **p*-value less than 0.05 is considered statistically significant. | | | | | |  |

| **Supplementary Table 2 Baseline characteristics and primary outcome by the recruitment site** | | | | |
| --- | --- | --- | --- | --- |
| **Characteristic** | | **Siriraj Hospital**  **(n = 341)** | **Bhumibol Adulyadej Hospital**  **(n = 59)** | ***p**** |
| Maternal age (year) | | 32.0 [27.0, 36.0]  (n=337) | 30.0 [26.0, 33.0]  (n=57) | 0.02* |
| Gestational age (weeks) | | 37.0 [36.0, 38.0] | 37.0 [35.0, 38.0] | 0.26 |
| Male sex | | 172 (50.4%) | 27 (45.8%) | 0.51 |
| Birth weight (g) | | 2,590.0  [2,280.0, 3,140.0] | 2,680.0  [2,372.0, 3,216.0] | 0.33 |
| Small-for-gestational age | | 42 (12.3%) | 10 (16.9%) | 0.33 |
| Large-for-gestational age | | 30 (8.8%) | 7 (11.9%) | 0.45 |
| Postnatal age at diagnosis of hypoglycemia (hour) | | 2.2 [2.0, 5.0] | 1.0 [1.0, 1.3] | <0.001* |
| Allocation group | |  |  |  |
|  | 50% dextrose group | 175 (51.3%) | 30 (50.8%) | 0.95 |
|  | Placebo | 166 (48.7%) | 29 (49.2%) |  |
| IV dextrose therapy | | 20 (5.9%) | 7 (11.9%) | 0.10 |
| Data are presented in number (percentage) or median [25^th^,75^th^ percentile].  **p*-value less than 0.05 is considered statistically significant. | | | | |

| **Supplementary Table 3 Model-predicted blood glucose increment and treatment difference by postnatal age depicted in hours** | | | | |
| --- | --- | --- | --- | --- |
| **Postnatal hours** | **Predicted ΔBG (Placebo) [95% CI]** | **Predicted ΔBG (50% Dextrose) [95% CI]** | **Treatment Difference [95% CI]** | ***p**** |
| 0 | 24.6 [21.9, 27.3] | 33.0 [30.2, 35.8] | +8.4 [4.5, 12.3] | <0.001* |
| 1 | 24.8 [22.3, 27.2] | 32.3 [29.8, 34.7] | +7.5 [4.0, 11.0] | <0.001* |
| 2 | 24.9 [22.7, 27.2] | 31.6 [29.3, 33.8] | +6.6 [3.5, 9.8] | <0.001* |
| 4 | 25.3 [23.2, 27.3] | 30.1 [28.1, 32.1] | +4.9 [2.0, 7.7] | <0.001* |
| 6 | 25.6 [23.4, 27.8] | 28.7 [26.5, 30.9] | +3.1 [0.0, 6.2] | 0.049* |
| 8 | 25.9 [23.3, 28.6] | 27.3 [24.6, 30.0] | +1.3 [-2.4, 5.1] | 0.483 |

ΔBG = change in blood glucose (BG) (mg/dL). Values represent Average Marginal Effects (AMEs), predicting the treatment outcome based on the actual empirical distribution of infant characteristics in the cohort. Predictions derived from the linear mixed-effects model presented in Table 3 (N = 444 hypoglycemic episodes across 398 infants). Representative postnatal ages are shown. *p*-values test the treatment difference (50% dextrose vs. placebo) at each postnatal age. **p*-value less than 0.05 is considered statistically significant.
